# Parental kinship coefficient but not paternal coloration predicts early offspring growth in lake char

**DOI:** 10.1038/s41437-024-00678-1

**Published:** 2024-03-13

**Authors:** Laura Garaud, David Nusbaumer, Lucas Marques da Cunha, Christian de Guttry, Laurie Ançay, Audrey Atherton, Emilien Lasne, Claus Wedekind

**Affiliations:** 1 Department of Ecology & Evolution, University of Lausanne, Lausanne, Switzerland; 2 Swiss Institute of Bioinformatics (SIB), Environmental Bioinformatic Group, Lausanne, Switzerland; 3 Université Savoie Mont Blanc, INRAE, UMR CARRTEL, Station d’Hydrobiologie Lacustre, Thonon Cedex, France; 4 UMR DECOD (Ecosystem Dynamics and Sustainability), INRAE, Institut Agro, IFREMER, Rennes, France

**Keywords:** Sexual selection, Inbreeding

## Abstract

The ‘good genes’ hypotheses of sexual selection predict that females prefer males with strong ornaments because they are in good health and vigor and can afford the costs of the ornaments. A key assumption of this concept is that male health and vigor are useful predictors of genetic quality and hence offspring performance. We tested this prediction in wild-caught lake char (*Salvelinus umbla*) whose breeding coloration is known to reveal aspects of male health. We first reanalyzed results from sperm competition trials in which embryos of known parenthood had been raised singly in either a stress- or non-stress environment. Paternal coloration did not correlate with any measures of offspring performance. However, offspring growth was reduced with higher kinship coefficients between the parents. To test the robustness of these first observations, we collected a new sample of wild males and females, used their gametes in a full-factorial in vitro breeding experiment, and singly raised about 3000 embryos in either a stress- or non-stress environment (stress induced by microbes). Again, paternal coloration did not predict offspring performance, while offspring growth was reduced with higher kinship between the parents. We conclude that, in lake char, the genetic benefits of mate choice would be strongest if females could recognize and avoid genetically related males, while male breeding colors may be more relevant in intra-sexual selection.

## Introduction

Females may get direct or indirect benefits when choosing their mate (Andersson 1994). Direct benefits include, e.g., nuptial gifts, protection, and paternal care. Indirect benefits increase offspring fitness through genetic effects. Arguably, the genetic consequences of mate choice are, therefore, best studied in species with external fertilization and little or no parental care (Neff and Pitcher 2005). Salmonid fish are excellent models in this context because they spawn externally and leave their embryos to develop, for example, in the interstices of gravel. Moreover, much is known about their ecology and life history. Experimental fertilizations and the rearing of large numbers of embryos, each in its own container, allow separating paternal from maternal effects on offspring phenotypes and testing for various types of interactions with the necessary numbers of independent replicates (e.g. Clark et al. 2014; Marques da Cunha et al. 2019).

Salmonid fish have polygamous mating systems that have been discussed as potentially influenced by intra-sexual dominance, endurance rivalry, scramble competition, sperm competition, and mate choice (Esteve 2005; Auld et al. 2019). Intra-sexual dominance is usually determined in fights or displays of fighting ability. Normally, larger males with well-developed secondary sexual characteristics win such dominance contests in salmonid fish (Jacob et al. 2007; Neff et al. 2008), non-salmonid fish (Jacob et al. 2009), and other animals (Andersson 1994). Male reproductive success is also dependent on how long they can remain reproductively active during the breeding season (endurance rivalry), how close they can position themselves to the vent of spawning females on their own and on other males’ spawning territories (scramble competition), and on their sperm number, motility, velocity, and longevity during simultaneous spawning with competitors (sperm competition). In comparison to these first four characteristics of spawning, female choice is often assumed to play a minor role in salmonid fish. However, females have frequently been observed to delay spawning when courted by non-desired males (Berejikian et al. 2000; Petersson and Jarvi 2001) or show aggressive behavior towards some types of males (Garner et al. 2010). As a consequence, female choice seems still possible in the salmonids (Esteve 2005; Auld et al. 2019).

Potential genetic benefits of mate choice can be grouped into two categories: additive genetic effects (‘good genes’) and non-additive genetic effects (‘compatible genes’) (Neff and Pitcher 2005; Achorn and Rosenthal 2020). Additive genetic effects are typically assumed to be revealed by a male’s health and vigor because only males in good health and vigor can afford the costs that are linked to extraordinary colors, morphological structures, or behaviors that would potentially make males sexually attractive (Andersson 1994). The correlation between such signals of attractiveness and the signaler’s health and vigor could theoretically be strong (Fry 2022) but is expected to vary, for example, because of age-specific signaling strategies in iteroparous species (Proulx et al. 2002). In fish, additive genetic benefits have indeed been found to be strong (Wedekind et al. 2001; Pitcher and Neff 2007; Kekäläinen et al. 2010) or weak (Wedekind et al. 2008; Houde et al. 2016) and are not sufficiently understood yet.

When mate choice is driven by non-additive genetic benefits, sexual signals need not be based on costly and conspicuous traits but can instead be non-handicapping signals such as, for example, body or urinary odors (Wedekind 1994). Non-additive genetic effects could be caused by various types of interactions that may include dominance effects or epistasis. Most examples of mate preference for genetic compatibility are about assortative mating in hybrid zones or about inbreeding avoidance (Tregenza and Wedell 2000). Inbreeding avoidance can be achieved in various ways, including mate choice based on genetic characteristics that would usually reveal kinship, for example, genes of the major histocompatibility complex (MHC) (Ruff et al. 2012; Davies 2013; Kamiya et al. 2014).

Here we compare the potential benefits if females were to mate with colorful males in good health and vigor (i.e., ‘good genes’ sexual selection) to the potential benefits of avoiding males with high kinship coefficient (i.e., a possible form of ‘compatible genes’ sexual selection). We use the lake char (*Salvelinus umbla*) as a model, and offspring growth before the onset of exogenous feeding as an indicator of genetic quality. After controlling for maternal environmental effects (egg size and egg content), such growth is best determined with several measures because, for example, the timing of hatching and the onset of exogenous feeding are only partly influenced by larval or yolk sac size and can also depend on personality traits and on how an individual perceives its environment (Wedekind and Müller 2005; Andersson et al. 2013; Pompini et al. 2013). Genetic effects on growth are then revealed, for example, if paternity affects hatchling size but not the timing of hatching, or if it affects yolk sac size but not larval length at a given point in time. Such laboratory data can be useful predictors of juvenile growth in the wild (Bylemans et al. in press).

The lake char is endemic to lakes in the Alpine region and closely related to the Arctic char (*S. alpinus*) (Kottelat and Freyhof 2007). While Arctic char often develop strong red breeding colorations in both sexes (Janhunen et al. 2011), male lake char of our study population mostly develop a yellow breeding coloration while females remain pale (Nusbaumer et al. 2023). In wild Arctic char, coloration tends to increase with body length (Figenschou et al. 2013; Johansen et al. 2019) and redder males have been observed to show higher plasma testosterone levels (Johansen et al. 2019), suffer less from infections (Johansen et al. 2019), and show lower lymphocyte counts (Skarstein and Folstad 1996). Wild lake char show similar patterns: more brightly colored males are typically larger and suffer less from relative lymphocytosis and thrombocytosis, two indicators of acute infections and hence of current health and vigor, than pale males (Nusbaumer et al. 2023). Breeding colors of wild-caught char therefore seem to fulfill key expectations for ‘good-genes’ indicators in sexual selection (Andersson 1994), but their potential role in mate choice and/or male-male competition is not sufficiently clear yet (Nusbaumer et al. 2023).

We first re-analyze sperm competition trials that resulted in embryos of known parenthood because of genetic paternity analyses (Nusbaumer et al. 2023). The experimental protocol of this first experiment allowed for the study of three different types of questions: First, the addition or no addition of ovarian fluids (after a first round of thorough washing of the eggs and before sperm were added) allowed for investigating the effects of ovarian fluids on sperm competition. These effects turned out to be significant (Nusbaumer et al. 2023). Second, after a less thorough washing of the freshly fertilized eggs, the remainder of the ovarian fluids on the eggs were expected to support microbial growth and hence induce stress to the embryos (Jacob et al. 2010; Wedekind et al. 2010). This allowed testing for potential sex-specific effects of stress and non-stress environments on embryo and larval development. Female embryos showed indeed lower stress tolerance than males (Nusbaumer et al. 2021). Here we focus on the third question that has not been addressed yet: We compare the embryos’ performance to their fathers’ coloration and the kinship coefficients between their parents. We then test the robustness of these results in a new full-factorial breeding experiment based on a new sample of males and females caught in the wild. The breeding design of this second experiment allows controlling for potentially confounding maternal environmental effects so that embryo and larval growth under stress and non-stress conditions are useful indicators of male genetic quality. We focus on the question of whether females would obtain indirect benefits from (i) preferring brightly colored males, and/or (ii) avoiding males with whom they share high kinship coefficients.

## Material and methods

### Sampling and determining phenotypes and genotypes

Wild lake char were caught in December 2017 (10 males and 4 females) and December 2018 (25 males and 8 females) from Lake Geneva in gill nets, anesthetized in a solution of 0.03% eugenol and ethanol at 1:9 ratio, photographed under standardized conditions, and stripped of their gametes. These gametes were then used for 2 different types of fertilization experiments as explained below.

Male breeding coloration (that is mostly yellow in the study population) was analyzed from the photos in Fiji as described in Nusbaumer et al. (2023). Briefly, the white balance was standardized based on the white and black sections of the color scale on each photo. Male yellowness was then measured as the b* components of the CIE-L*a*b* color space model (León et al. 2006), where the L* axis quantifies lightness from dark to light, the a* axis ranges from green to red, and the b* axis from blue to yellow, with high b* values indicating strong yellow color (the axes scale from −100 to 100). After transformation of the image in a Lab-Stack with the function run (“Lab Stack”), all CIE-L*a*b* color components were measured from 3 squares in the pectoral region, the ventral region, and the anal region (Nusbaumer et al. 2023) and then averaged. Lightness and redness were significantly correlated with yellowness (the yellower the lighter and/or the redder (Nusbaumer et al. 2023)) and were therefore ignored for further analyses.

The DNAeasy Blood & Tissue kit (Qiagen, Hilden, Germany) was used to extract DNA from up to 20 mg of fin samples (with an extraction robot, following manufacturer’s instructions). DNA concentration was measured using Qubit 2.0 (Thermo Fisher Scientific, Waltham, Massachusetts) while its integrity was verified on a 1% agarose gel. The DNA of each individual was standardized to a concentration of 20 ng/µl. The libraries were prepared as follows: the enzymes *Eco*RI-HF and *Msp*I (New England Biolab, Ipswich MA, USA) were used for DNA digestion and a unique *Eco*RI barcode was ligated to each individual (Brelsford et al. 2016). After library purification, PCR amplification was performed, and fragments were size-selected between 400–550 bp. The parents of the first experiment were then single-end genotyped on 2 lanes with fragments of 110 bp length, and the parents of the second experiment on one lane of Illumina HiSeq 2500 with fragments of 150 bp length at Lausanne Genomic Technologies Facility (University of Lausanne).

After quality control with FASTQC v0.11.7, the resulting ddRAD sequencing data of the two experiments were analyzed separately using Stacks (Catchen et al. 2013) with the default parameters unless otherwise specified. The maximum number of alleles at a single de novo locus was set by default at 2 to detect putative duplicated loci. Individual sequences were demultiplexed using *process_radtags* (Catchen et al. 2013) in the 1^st^ experiment (see also Nusbaumer et al. 2023) and trimmomatic (Bolger et al. 2014) in the 2^nd^ experiment and trimmed to 110 bp before mapping to the *Salvelinus spp*. reference genome (NCBI accession number SRP101753) using BWA (Li and Durbin 2010). The resulting bam files were sorted using *samtools* (Li et al. 2009) and processed using *gstack* (Stacks 2.53). *Populations* was used to generate the VCF file considering only loci present in at least 80% of the individuals (–*r* 0.8) and marker heterozygosity of 0.5 (*–max-obs-het* 0.5). To reduce incorrect heterozygosity call and remove paralogs, *vcftools* (Danecek et al. 2011) was used for filtering the loci for coverage between 10X (*–min-meanDP* 10) and 50X (–*max-meanDP* 50) for the 1^st^ experiment and between 7X (*–minDP* 7), and 60X (*–maxDP* 60) in the 2^nd^ experiment, presence in all individuals (*–max-missing* 1), and at Hardy-Weinberg equilibrium with a significance threshold of 0.05 (*–hwe* 0.05). The difference in coverages was due to the differences in sequencing methodologies and data qualities (thresholds were selected based on correlation analyses between coverage and heterozygosity). This led to a total of 4,150 SNPs with a mean coverage of 29X for all individuals of the 1^st^ experiment, and a total of 975 SNPs with a mean coverage of 27X in 30 of the 33 individuals of the 2018 samples. Three males with low genotyping rate (>30% missing data) were excluded from all further analyses.

The difference in SNP counts between the two experiments can be attributed to different sequencing strategies. A deep coverage sequencing strategy was employed for individuals in the first experiment to enhance data quality, while individuals in the second experiment underwent routine sequencing as a confirmation of the initial experiment’s findings. Both numbers of SNPs have been demonstrated to provide reliable estimates of relatedness and multi-locus heterozygosity (MLH) (Kopps et al. 2015; Kardos et al. 2018).

The kinship coefficient between males and females was estimated with the *beta.dosage* function in Hierfstat 0.04-30 (Goudet 2005). This function generates a genomic matrix based on allele dosage that allows estimates of the kinship coefficients based on the proportion of shared alleles (Goudet et al. 2018). These kinship coefficients correspond to the expected average inbreeding coefficient of the progeny within a family (µF_β.offspring_). Individual inbreeding coefficients (F_β.parent_) were then obtained by extracting the diagonal of the genomic matrix.

### Experiment 1

The first experiment was done with the 14 lake char that were sampled in 2017. The experimental procedures had been optimized to test for the potential effects of ovarian fluids in sperm competition. The results of the corresponding analyses are published in Nusbaumer et al. (2023). The experiment also allowed us to test whether embryo stress tolerance is sex-specific as reported in Nusbaumer et al. (2021). Here, these data are re-analyzed to study the potential genetic benefits of different mate choice scenarios.

The experimental procedures are described in detail in Nusbaumer et al. (2021, 2023). Briefly, milt was stripped from the males into individual containers, mixed 1:9 with diluted Storefish© (IMV Technologies, France; here diluted with 1:9 with MilliQ water), and stored on ice. Eggs were stripped from females into individual plastic containers. Ovarian fluid was separated from the eggs with a syringe and stored at 4 °C. The eggs were then washed twice with 200 mL Ovafish (IMV Technologies, France). Diluted milt of 2 males each (haphazardly assigned from the 10 males) were mixed such that each male was represented with 25 million active sperm per mL of the mix. One mL of such a mix was then used to fertilize 24 eggs of a female in wells of 6-well plates (Falcon, BD Biosciences, Allschwil, Switzerland). This 1 mL of the mix was activated in a separated tube with either 4 mL of chemically standardized water (i.e. reconstituted according to the OECD guidelines, OECD 1992) or 4 mL of standardized water with ovarian fluid (ratio ovarian fluid to water = 1:2), vortexed for 5 s, and immediately poured into a well with the eggs. Two minutes later, 16.8 mL of standardized water was added to fill the wells. The eggs were then left undisturbed for 2 h of egg hardening. Each mix of sperm was used full-factorial, fully balanced, and repeated with or without ovarian fluids on the eggs of all females, resulting in a total of 80 batches of 24 eggs each (5 types of sperm mixes ×4 females ×2 experimental conditions ×2 replicates = 80 batches).

Ten of these 80 batches of eggs were accidentally lost (all from the same female and exposed to sperm activated in water only), reducing the number of eggs to be monitored to *N* = 1680. These freshly fertilized eggs were batch-wise rinsed in a sterilized tea strainer under cold tap water for 30 s (4 L/min), then distributed singly to 24-well plates (each embryo in its own compartment filled with 1.8 mL autoclaved standardized water) and raised at 4.5 °C (von Siebenthal et al. 2009). The rinsing of the freshly fertilized eggs was considered necessary to avoid high embryo mortalities (Wedekind and Müller 2004) but it was done such that remainders of ovarian fluids could still be expected on the developing eggs (Nusbaumer et al. 2021). The rate of embryonated eggs was determined at 28 dpf (days past fertilization). Towards the end of the embryo stage, all wells were checked daily to record embryo mortality and hatching dates. Freshly hatched larvae were immediately transferred to new 5 mL wells with standardized water only. Standard photos were taken on the same day to measure larval length in ImageJ (Supplementary Fig. S1a). Larval length and yolk sac volumes were measured on the day of hatching and 14 days later (Supplementary Fig. S1b) when larvae were humanely killed (with an anesthetic overdose) and genotyped for 3 to 6 microsatellite markers to identify their fathers (details in Nusbaumer et al. 2021). All embryos and larvae that had died before were also genotyped. A sex-specific marker was added to the first multiplex of 3 microsatellite markers to test for the sex-specific effects on embryo development (Nusbaumer et al. 2021). Because these sex-specific effects turned out to be sublethal (final sex ratio = 49.96% males) and not family-specific (Nusbaumer et al. 2021), offspring sex was ignored for the present analyses.

### Experiment 2

The gametes of the 25 males and 8 females of the 2018 sample were stripped into individual containers as in the first experiment. The eggs of each female were about equally distributed to 25 Petri dishes (Greiner Bio-One, Austria). Undiluted milt of one male each was added to the individual batches of eggs to produce all possible 200 families (8 females x 25 males). Standardized water was then added to activate the sperm and induce fertilization. After letting the freshly fertilized eggs harden for 2 h, 72 eggs per sib group (14,400 in total) were transported on ice to a climate chamber where they were batch-wise rinsed in cold tap water (in sterilized tea strainers for 30 seconds at 2 L/min) and singly distributed to 24-well plates for two separate experiments: 24 eggs/sib group were used for the present study and the remaining 48 eggs/sib group for a study on effects of a common chemical pollutant (Garaud et al. in preparation). The rate of embryonated eggs was determined at 35 dpf.

At 69 dpf, i.e. about three weeks before hatching (Supplementary Fig. S2), half of the eggs per sib group were exposed to the bacterium *Aeromonas salmonicida* while the other half was sham-treated. This treatment was prepared as follows: Dry-frozen *A. salmonicida* that had previously been isolated from brown trout (*Salmo trutta*) were rehydrated and inoculated at 22 °C for 24 h in TBS (Tryptic Soy Broth, Fluka, Switzerland). Cultures were then washed and counted in a Helber counting chamber. Bacteria were diluted in autoclaved standardized water and 1% TSB such that adding 100 µL in each well plus 100 µL MilliQ water would result in 0.5 × 10^6^ cfu/mL in the well. The sham-treated embryos received 200 µL standardized water. The bacterial density was chosen to be likely sublethal (D. Nusbaumer, unpublished observations) so that the more informative indicators of embryo development rate could be used as a dependent variable instead of the binary embryo mortality. A control for the potential effects of TSB was not added because addition of bacteria and TSB were only used here to induce microbial stress.

Towards the expected start of hatching, all wells were checked daily to record embryo mortality and hatching date. Freshly hatched larvae were immediately transferred to new 5 mL wells with standardized water only. Standard photos were taken on the same day to determine larval length in ImageJ.

Because larval size measurements turned out to be statistically linked to the timing of hatching (see Results), larval length was again measured 14, 28, and 42 dph (days past hatching) while yolk sac volume was determined on the day of hatching and at 42 dph. From these measurements, larval length at 130 dpf (L_130dpf_), a haphazardly chosen time point that was defined by the time of fertilization (instead of the time of hatching) to obtain a standardized measurement of larval size (Supplementary Figs. S1, S2) was calculated as

1 $${{\rm{L}}}_{130{\rm{dpf}}}={{\rm{L}}}_{{\rm{hatching}}}+(130-{{\rm{D}}}_{{\rm{hatching}}})({{\rm{L}}}_{42{\rm{dph}}}-{{\rm{L}}}_{{\rm{hatching}}})/42$$

where L_hatching_ is the larval length at hatching, D_hatching_ is the number of days from fertilization to hatching, and L_42dph_ is the larval length at 42 dph. Yolk sac volume at 130 dpf was determined as

2 $${{\rm{YS}}}_{130{\rm{dpf}}}={{\rm{YS}}}_{{\rm{hatching}}}+(130-{{\rm{D}}}_{{\rm{hatching}}})({{\rm{YS}}}_{42{\rm{dph}}}-{{\rm{YS}}}_{{\rm{hatching}}})/42$$

where YS_hatching_ is the yolk sac volume at hatching and YS_42dph_ the yolk sac volume at 42 dph. After the last size measurements, larvae were humanely killed (like in the 1^st^ experiment) and stored in 70% ethanol.

### Statistics

Statistical analyses were done in JMP® Pro 17.0.0 (JMP® 1989–2023). Estimates of variance components were based on the restricted maximum likelihood methods (REML, with unbounded variance components) applied to linear mixed models (LMM). Wald random effects tests were used to test the random effects (Snijders and Bosker 2012). In experiment 1, LMMs were used to predict hatching time, larval length, and yolk-sac volume at hatching and 14 days post-hatching, with treatment as a fixed factor, hatching date (when predicting larval length and yolk-sac volume), male yellowness, and the kinship coefficient between males and females as covariates, and family identity and their interactions with treatments as random factors. The focus of these LMMs is on the effects of male yellowness and parental kinship while family and treatment effects had been reported in Nusbaumer et al. (2021) and needed to be controlled here.

In experiment 2, LMMs based on REML were first used to predict hatching time, larval length, and yolk-sac volume at hatching, with the microbial treatment as a fixed factor. If hatchling size measures were the dependent variables, the hatching date was included as a covariate. In the first set of LMMs, dam, sire, dam x sire, and all possible interactions to treatment were added as random factors before stepwise removal of non-significant interactions. The model was then simplified by replacing the factors dam and sire with the new random factor “family” and adding male yellowness and/or the kinship coefficient between the parents of each family as covariates.

## Results

### Inbreeding coefficients and skin coloration

**Fig. 1:**
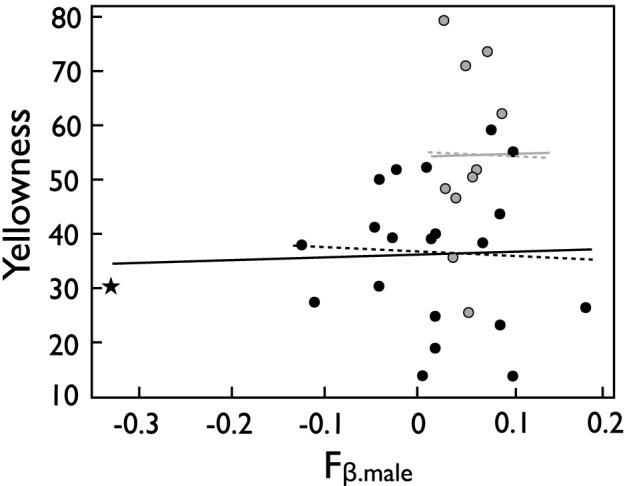
Male skin coloration (yellowness) predicted by the males’ inbreeding coefficients (F_β.male_). Gray symbols and gray lines represent the 10 males of the 1^st^ experiment (replotted from Fig. 1b in Nusbaumer et al. 2023). Black symbols and black lines represent the 22 males of the 2^nd^ experiment. The non-hatched lines give the fits from a multiple regression after the removal of the non-significant interaction term, the hatched lines give the analogous fits after removal of the males with the very low F_β_ value (marked with a star). See text for statistics.

The males’ yellowness could not be predicted from their inbreeding coefficients in either year, while the males used in the 1^st^ experiment turned out to be more intensely colored than the males in the 2^nd^ experiment (Fig. 1; multiple regression, effects of F_β.parent_: F = 0.04, *p* = 0.85; year: F = 9.6, *p* = 0.005).

### Experiment 1

**Fig. 2:**
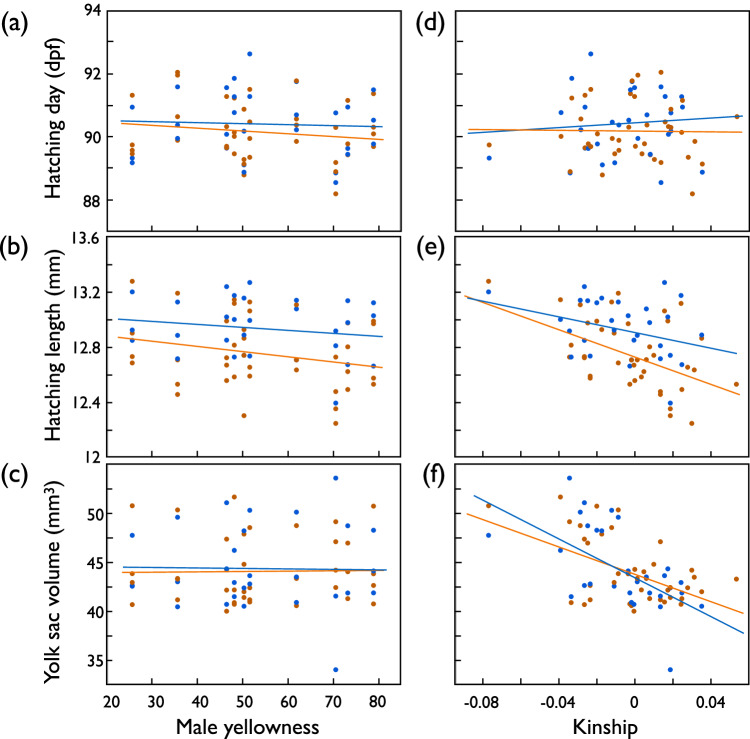
Offspring growth predicted by paternal coloration or parental kinship coefficient in the first experiment (fertilization during sperm competition). Hatching time (dpf, days past fertilization), hatchling length, and yolk sac volume of hatchlings (means per full-sib family) versus (**a**–**c**) paternal skin coloration (“male yellowness”), (**d**–**f**) the parental kinship coefficient (“kinship”) after exposure to organic pollution (orange symbols and regression lines), or not exposed (blue). See Table 1 for statistics.

Table 1 gives the effects of paternal yellowness and the parental kinship coefficient on measures of offspring growth while controlling for the family and treatment effects that have been reported before (Nusbaumer et al. 2021). Paternal yellowness did not significantly predict any offspring characteristics (Table 1; Supplementary Table S1; Fig. 2a–c, Supplementary Fig. S3a, b). However, the parental kinship coefficient was a strong predictor of embryo and larval growth: With higher kinship, the larvae hatched at smaller lengths and with smaller yolk sacs despite hatching at similar times (Table 1; Fig. 2d–f). Fourteen days after hatching, the effects of kinship on larval length were no more statistically significant, but yolk sac volumes were still smaller with higher kinship (Supplementary Table S1; Supplementary Fig. S3c, d). None of the pairwise interactions between treatment, yellowness, and kinship were significant (Table 1).

**Table 1:** First experiment: Linear mixed model on embryo hatching time, hatchling length, and yolk sac volume at the day of hatching when predicted by treatment (support of microbial symbionts by remainders of ovarian fluid) and paternal skin coloration (yellowness) and/or the parental kinship.

Effects	Hatching time				Hatchling length				Yolk sac volume			
Fixed effects	Coefficients	DF	*t*	*p*	Coefficients	DF	*t*	*p*	Coefficients	DF	*t*	*p*
Intercept	90.59 ± 0.55	36.45	166.1	<0.001	8.67 ± 0.73	1192	11.9	<0.001	17.85 ± 10.14	1222	1.76	0.08
Treatment	0.04 ± 0.06	32.65	0.55	0.59	0.07 ± 0.01	24.01	4.50	<0.001	0.03 ± 0.21	22.83	0.13	0.90
Yellowness	−0.01 ± 0.01	36.17	−0.76	0.45	−0.002 ± 0.002	36.13	−0.80	0.43	0.01 ± 0.03	34.84	0.29	0.78
Kinship	−2.37 ± 6.67	38.22	−0.35	0.72	−3.93 ± 1.55	38.76	−2.54	**0.015**	−83.89 ± 20.79	37.72	−4.04	**<0.001**
Yellowness × kinship	−0.27 ± 0.36	37.55	−0.77	0.45	−0.04 ± 0.08	38.3	−0.45	0.65	−1.37 ± 1.11	37.3	−1.23	0.23
Treatment × yellowness	0.002 ± 0.004	30.32	0.61	0.55	0.0003 ± 0.001	21.45	0.44	0.66	−0.0002 ± 0.01	20.53	−0.01	0.99
Treatment × kinship	−0.60 ± 2.69	36.65	−0.22	0.82	0.99 ± 0.61	27.6	1.61	0.12	0.90 ± 8.79	26.73	0.10	0.92
Hatching time					0.05 ± 0.008	1275	5.94	<0.001	0.28 ± 0.11	1302	2.56	0.01
Random effects^a^	Variance components				Variance components				Variance components			
Family ID	0.73 ± 0.21			<0.001	0.04 ± 0.01			<0.001	6.87 ± 2.07			<0.001
Family × treatment	0.12 ± 0.06			0.04	0.003 ± 0.003			0.37	0.61 ± 0.72			0.40
Residual	2.18 ± 0.09				0.16 ± 0.01				35.0 ± 1.39			

Hatching time was included as a further covariate when testing for effects on hatchling length and yolk sac volume. The table gives the coefficients (±standard error), the denominator degrees of freedom (DF, based on the Kenward–Roger first-order approximation; the numerator degrees of freedom is always 1), the t values (the ratio of the coefficient to its standard error), and the corresponding two-tailed *p*-values. Family identity and the interaction between family ID and treatment were included as random factors. New significant *p*-values are highlighted in bold (parental effects and effects of treatment and hatching date had already been reported in Nusbaumer et al. (2021)).

^a^REML unbounded variance components ± standard error, Wald *p*-values.

### Experiment 2

The overall rate of embryonated eggs at 35 dpf (before treatment was applied) was 81.0% and varied between females (from 42.2% to 92.7%; χ^2^ = 516.2, df = 7, *p* < 0.001) and males (χ^2^ = 68.3, df = 20, *p* < 0.001). This rate could not be predicted by male yellowness (r = 0.05, *n* = 21, *p* = 0.83) nor by the mean genetic distance between a male to all females (mean kinship per male; r = 0.02, *n* = 21, *p* = 0.93). Embryo mortality from 35 dpf until the day of hatching was 0.9% and not linked to treatment (χ^2^ = 0.12, df = 1, *p* = 0.73).

Exposure to microbes reduced embryo and larval growth because it not only led to earlier hatching of smaller larvae, but these larvae were also smaller than sham-exposed ones at 130 dpf, i.e., at a late larval stage that was defined by time since fertilization instead of the time of hatching (Table 2; Fig. 2). Embryo and larval growth were significantly influenced by sire and dam x sire interaction effects while dam main effects were not significant (Table 2). There was also a dam x sire x treatment interaction effect on the time of hatching, while no such interaction between parents or parental combinations with treatment was found for embryo or larval size measures (Table 2).

**Table 2:** Second experiment: Linear mixed model on embryo hatching time, hatchling length, and yolk sac volume at hatching when predicted by treatment (exposure to *Aeromonas salmonicida*; fixed effect) and dam, sire, dam x sire, and their interaction with treatment (random factors).

Effects	Hatching time				Hatchling length				Larval length 130dpf				Yolk sac volume 130dpf			
Fixed effects	Coefficients	DF	*t*	*p*	Coefficients	DF	*t*	*p*	Coefficients	DF	*t*	*p*	Coefficients	DF	*t*	*p*
Intercept	93.39 ± 0.43	9.44	218.3	**<0.001**	7.07 ± 0.31	1836	23.0	**<0.001**	21.18 ± 0.40	1154	52.5	**<0.001**	−27.20 ± 4.16	1452	−6.53	**<0.001**
Treatment	−0.33 ± 0.10	8.45	−3.34	**0.01**	−0.10 ± 0.007	2997	−14.5	**<0.001**	−0.10 ± 0.01	2399	−11.3	**<0.001**	0.05 ± 0.10	2709	0.50	0.62
Hatching time					0.06 ± 0.003	2692	18.0	**<0.001**	−0.05 ± 0.004	2086	−12.2	**<0.001**	0.61 ± 0.04	1920	13.84	**<0.001**
Random effects^a^	Variance components				Variance components				Variance components				Variance components			
Dam	1.17 ± 0.67			0.08	0.025 ± 0.014			0.07	0.07 ± 0.04			0.07	4.61 ± 2.51			0.07
Sire	0.55 ± 0.23			**0.01**	0.007 ± 0.003			**0.008**	0.01 ± 0.004			**0.007**	0.27 ± 0.17			0.12
Dam × sire	0.38 ± 0.15			**0.01**	0.004 ± 0.002			**0.004**	0.004 ± 0.002			0.06	0.62 ± 0.25			**0.015**
Dam × treatment	0.07 ± 0.07			0.33												
Sire× treatment	0.07 ± 0.08			0.40												
Dam × sire × treatment	0.88 ± 0.17			**<0.001**												
Residual	4.13 ± 0.11				0.158 ± 0.004				0.20 ± 0.006				24.5 ± 0.68			

Hatching time was included as a further covariate when testing for effects on hatchling and larval lengths and yolk sac volume. The table gives the parameters as in Table 1 and after the stepwise removal of non-significant interaction terms. Significant *p*-values are highlighted in bold.

^a^REML unbounded variance components ± standard error, Wald *p*-values.

The significant parental effects on hatching time and embryo and larval growth were confirmed when summarized in a new factor “family” (Table 3). The mean hatching date per family was again treatment dependent while hatchling length and larval size at 130 dpf were not (family x treatment interactions in Table 3). All these results were confirmed in analogous models that excluded the one male with the extra-ordinary low F_β.male_ (Supplementary Table S2).

**Table 3:** Second experiment: Linear mixed model on embryo hatching time, hatchling length, larval length, and yolk sac volume at 130 dpf when predicted by treatment (exposure to *Aeromonas salmonicida*) and paternal skin coloration (yellowness) and/or parental kinship.

Effects	Hatching time				Hatchling length				Larval length 130dpf				Yolk sac volume 130dpf			
Fixed effects	Coefficients	DF	*t*	*p*	Coefficients	DF	*t*	*p*	Coefficients	DF	*t*	*p*	Coefficients	DF	*t*	*p*
Intercept	93.74 ± 0.40	172.7	235.5	**<0.001**	7.15 ± 0.32	2614	22.6	**<0.001**	21.52 ± 0.42	2254	50.9	**<0.001**	−25.89 ± 4.34	2417	−5.97	**<0.001**
Treatment	−0.32 ± 0.07	161.8	−4.89	**<0.001**	−0.10 ± 0.008	165.6	−13.2	**<0.001**	−0.10 ± 0.01	168.6	−10.8	**<0.001**	0.02 ± 0.11	164.1	0.20	0.84
Yellowness	−0.01 ± 0.01	172.3	−1.16	0.25	−0.0003 ± 0.001	169.9	−0.17	0.86	0.002 ± 0.002	168.8	0.90	0.37	0.02 ± 0.02	173.2	1.03	0.31
Kinship	−7.30 ± 2.40	170	−3.04	**0.003**	−0.33 ± 0.33	167.2	−0.98	0.33	−0.70 ± 0.46	164.4	−1.51	0.13	−11.65 ± 4.00	170.5	−2.92	**0.004**
Yellowness × kinship	0.46 ± 0.28	173.3	1.67	0.10	0.01 ± 0.04	170.4	0.33	0.74	0.02 ± 0.05	168.6	0.30	0.76	−0.53 ± 0.46	173.4	−1.15	0.25
Treatment ×yellowness	−0.007 ± 0.005	160.7	−1.28	0.20	0.002 ± 0.001	160	2.81	**0.006**	0.0001 ± 0.0001	161.5	0.13	0.90	0.01 ± 0.01	156.5	1.27	0.21
Treatment × kinship	0.62 ± 1.14	152.8	0.54	0.59	−0.13 ± 0.13	145.2	−1.00	0.32	−0.04 ± 0.16	142.7	−0.25	0.80	1.31 ± 1.82	144.8	0.72	0.47
Hatching time					0.06 ± 0.003	2745	17.3	**<0.001**	−0.06 ± 0.004	2299	−12.6	**<0.001**	0.58 ± 0.05	2513	12.79	**<0.001**
Random effects^a^	Variance components				Variance components				Variance components				Variance components			
Family ID	1.62 ± 0.27			**<0.001**	0.03 ± 0.005			**<0.001**	0.07 ± 0.01			**<0.001**	4.39 ± 0.72			**<0.001**
Family × treatment	1.01 ± 0.16			**<0.001**	0.003 ± 0.002			0.14	0.003 ± 0.003			0.40	0.80 ± 0.43			0.06
Residual	4.13 ± 0.11				0.16 ± 0.004				0.20 ± 0.006				24.11 ± 0.69			

Hatching time was included as a further covariate when testing for effects on hatchling length. The table gives the parameters as in Table 1. Family identity and the interaction between family ID and treatment were included as random factors. Significant *p*-values are highlighted in bold.

^a^REML unbounded variance components ± standard error, Wald *p*-values.

**Fig. 3:**
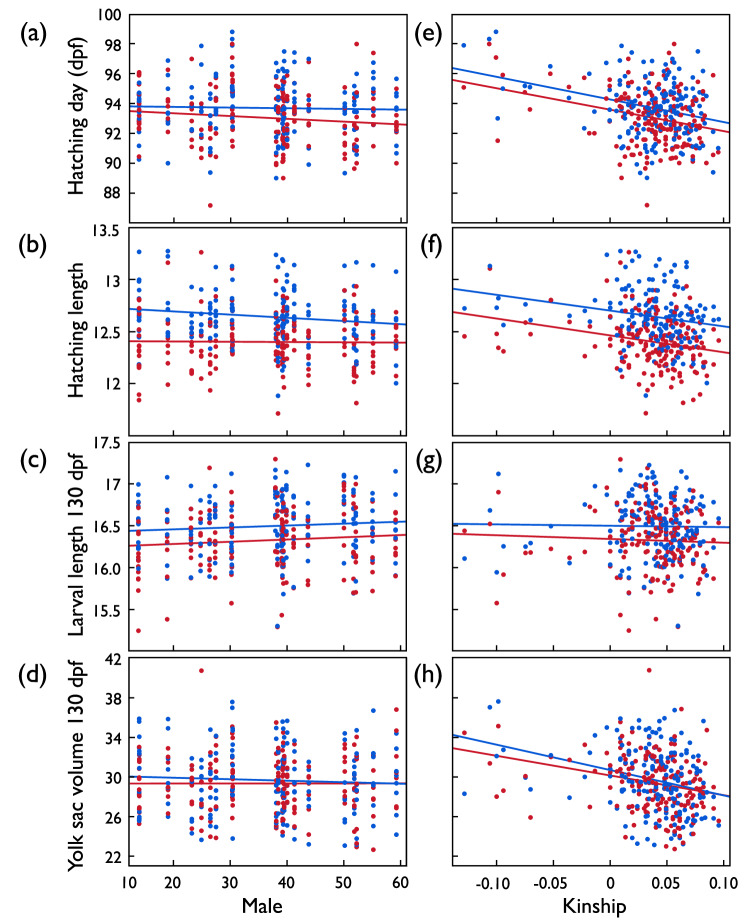
Second experiment: Embryo development after exposure to *Aeromonas salmonicida* (red symbols and regression lines) or sham exposure (blue). **a** Mean hatching time (dpf = days past fertilization), **b** mean larval length at hatching (mm), **c** mean larval length 130 dpf (mm), and **d** mean yolk sac volume 130 dpf (mm^3^) per full-sib family predicted by paternal skin coloration (“male yellowness”), and (**e**–**h**) predicted by the paternal kinship coefficient (“kinship”). See Table 3 for statistics.

Paternal yellowness did again not predict embryo growth: Neither the timing of hatching, nor hatching length or larval length at 130 dpf were correlated to paternal yellowness (Table 3; Fig. 2a–d). A significant interaction term between treatment and yellowness (Table 3) suggested that offspring of yellower males tend to hatch at smaller sizes when not stressed while there seemed to be no such link to male yellowness when embryos were raised under stress conditions (Fig. 3b). However, this interaction could not be confirmed at 130 dpf (Table 3; Fig. 2c, d). All these results were confirmed in analogous models that excluded the one male with the extra-ordinary low F_β.male_ (Supplementary Table S2; Supplementary Fig. S4).

The parental kinship coefficient was again a strong predictor of embryo and larval growth: With higher kinship, the larvae hatched earlier (Table 3; Fig. 2e), and while the effects of kinship on larval length at hatching or 130 dpf were not significant, yolk-sac volume at 130 dpf was significantly reduced with higher parental kinship (Table 3; Fig. 2f–h). These kinship effects on embryo and larval development were similar in both treatment groups (non-significant interaction terms in Table 3). All these results were confirmed in the model that excluded the male with the extra-ordinary low F_β.male_, except that parental kinship significantly affected larval size at 130 dpf while its effect on hatchling time was no more significant in the reduced model (Supplementary Table S2; Supplementary Fig. S4).

## Discussion

Female choice in salmonid fish is not sufficiently understood. The role of sexual displays is still unclear, and it is not fully solved whether salmonids can recognize and avoid genetically similar mates to reduce inbreeding in the next generation. Here we focused on the potential fitness benefits that lake char females would get if they were able to choose based on different types of information about males. Because their mating system arguably excludes direct benefits, we asked what type of genetic benefits were possible if females based their choice on male sexual displays that reveal aspects of male health (Nusbaumer et al. 2023) or on information about their genetic distance to a male (the kinship coefficient). It is still unclear what type of signals would reveal kinship in lake char. However, MHC-linked odors have been demonstrated to be important in the social communication of many vertebrates, including various salmonids and other fishes (Ruff et al. 2012). Such odors would reveal useful information about kinship within natural populations (Ruff et al. 2012).

We first re-analyzed results from an experiment whose design was optimized for studying other types of questions (Nusbaumer et al. 2021; Nusbaumer et al. 2023) and that resulted in embryos of known parentship. This first experiment therefore allowed us to test whether offspring performance could be predicted by male coloration or by the kinship coefficient between the parents. It turned out that male breeding coloration was not a useful predictor of offspring performance. However, the kinship coefficient correlated strongly with offspring performance: higher kinship led to reduced offspring growth.

We then collected a new and larger sample of males and females from the wild to specifically test for the robustness of these first results. We used the powerful full-factorial in vitro breeding (North-Carolina II design) and single rearing of a large number of offspring to separate dam, sire, and dam x sire effects on various aspects of offspring life history and growth. We found that the rate of embryonated eggs was not correlated to male yellowness or the kinship coefficients between the parents. Embryo mortality as determined from 35 dpf on could be ignored in our study because it was below 1%. We could therefore focus on the timing of hatching and the various growth indicators as measures of offspring performance. We found significant sire effects that, given our breeding design, reveal additive genetic effects on embryo and larval growth. Assuming that offspring growth is a good measure of genetic quality, we conclude from this first observation that males differ in their genetic quality. However, we found again no correlation between offspring growth and male yellowness. The observed variance in genetic quality was not revealed by male breeding coloration. We also found significant dam x sire interaction effects that revealed non-additive genetic effects on various aspects of offspring performance. Offspring performance hence depended on who was mated with whom in our full-factorial breeding experiment. When we then tested whether kinship between the parents predicted offspring life history and growth, we found that offspring performance improved with decreasing kinship coefficients. The results of our first experiment were therefore confirmed even though the calculation of the kinship coefficients was based on a lower number of SNPs in the second than in the first experiment.

The quantitative effects of parental kinship on embryo and larval growth remain to be more accurately determined. The protocol we used (Goudet et al. 2018) only provides the kinship of a pair of individuals relative to the average kinships in the population, i.e. the scale we used is sample-specific or population-specific at best. Moreover, we measured growth with different variables (hatching day, larval size, and yolk sac volume at different time points). This was necessary because it is, for example, possible that individuals of different sizes hatch at about the same time (Nusbaumer et al. 2021) or that individuals of similar sizes hatch at different times (Marques da Cunha et al. 2019). Differences in growth are then only seen when the various variables are measured and analyzed in parallel. It has not been solved yet how to best combine them into one growth parameter.

A study on possible genetic benefits is ideally based on potential fitness indicators, but fitness is notoriously difficult to measure. Here we focused on embryo and larval growth under stress and non-stress conditions, keeping the environmental stress at a sub-lethal level to avoid mortalities and to focus on the continuous and hence more informative measures of growth. Bylemans et al. (in press) recently tested the ecological relevance of such growth measurements in another salmonid and found that they were good predictors of juvenile size after their first 6 months in the wild. Juvenile size has long been known to positively affect survival and reproduction (Garcia de Leaniz et al. 2007). Embryo and larval growth are therefore useful indicators of offspring fitness. We conclude that if females could choose their mate, they would profit most from avoiding genetic similarity while ignoring male coloration to achieve the highest genetic benefit from mate choice. Analogous negative effects of genetic similarity were recently demonstrated in an amphibian (Byrne et al. 2021), and analogous non-significant effects of male coloration on offspring viability were recently reported for three-spined stickleback (*Gasterosteus aculeatus*) (Chiara et al. 2022).

It has been suggested that the ‘good genes’ hypothesis may be better described as the ‘absence of bad genes’ because condition-dependent ornaments may reveal the absence of deleterious mutations (Tomkins et al. 2004; Baur and Berger 2020). Lake char breeding coloration seems to be a condition-dependent sexual signal because it reveals aspects of male health (Nusbaumer et al. 2023). However, we found no correlation between male inbreeding coefficients and their coloration. This suggests that the variance in inbreeding coefficients among the males did not significantly affect the condition that their skin coloration reveals, for example, because acute infections may often be more relevant for condition-dependent traits than inbreeding coefficients.

Apart from revealing indicators of acute health, male yellowness is also positively linked to male size and measures of milt quality (Nusbaumer et al. 2023) that may include the quality of seminal fluids (Bartlett et al. 2017). Male coloration in lake char may therefore first be an indicator of current fighting ability (or willingness to fight) and hence of dominance in male-male competition (Johnstone 1997). In salmonids, the capacity of a male to dominate others seems to be an important predictor of male mating success in the wild (Auld et al. 2019). Our findings suggest, however, that females get little genetic benefit from mating with such males. This supports analogous results from breeding experiments with migratory and non-migratory brown trout (Jacob et al. 2007). Females may benefit from the protection that dominant males sometimes provide against egg predation during the first minutes after spawning (Frye et al. 2021) or from higher fertilization success due to milt and sperm traits linked to male dominance (Masvaer et al. 2004; Nusbaumer et al. 2023). However, if females aim for genetic benefits, we predict from our results that they should prefer genetically dissimilar males with whom they would produce offspring of low inbreeding coefficients. Females could, for example, increase the rate of mating with genetically dissimilar males by actively preferring dissimilar phenotypes (Gil et al. 2016) or by increasing the rate of spawning with an increasing number of males joining a dominant male for multi-male spawning (Petersson and Jarvi 2001).

Salmonid fish are famous for their olfactory ability (Keefer and Caudill 2014), and juvenile Arctic char learn to discriminate siblings by their MHC and other factors (Olsen et al. 2002). It is therefore possible that odors reveal alleles of the MHC via peptide ligands that are used as markers for the degree of kinship (Milinski et al. 2005). MHC-linked mate preferences could then be used to avoid genetically similar males (Ruff et al. 2012). It remains to be tested whether MHC-linked mate preferences can lead to inbreeding avoidance in lake char and hence to the genetic benefits we observed (Landry et al. 2001).

In conclusion, we found that male yellowness does not predict offspring survival, growth, or stress resistance. This secondary sexual trait of lake char does therefore not seem to be an indicator of ‘good genes’. Females could, however, significantly increase offspring growth by avoiding genetically similar males. The potential genetic benefits of mate choice would be large if females aimed for such non-additive genetic effects (‘compatible genes’) instead of potential additive genetic effects (‘good genes’).

### Supplementary information

Supplementary Material

## Data Availability

All data have been deposited on the Dryad repository: Experiment 1 (de Guttry et al. 2022; Nusbaumer et al. 2022), and Experiment 2 (Garaud et al. 2024).
