# Supplementary material for: Parental kinship coefficient but not paternal coloration predicts early offspring growth in lake char
Source: Heredity (Edinb). 2024 Mar 13;132(5):247–56. doi: 10.1038/s41437-024-00678-1 (PMC11074127; doi:10.1038/s41437-024-00678-1)
Supplement: Supplementary file 1 — Supplementary Material [file 41437_2024_678_MOESM1_ESM.docx]

***Supplementary information***

**Parental kinship coefficient but not paternal coloration predicts early offspring growth in lake char**

Laura Garaud^1^#, David Nusbaumer^1^#, Lucas Marques da Cunha^1^, Christian de Guttry^1,2^, Laurie Ançay^1^, Audrey Atherton^1^, Emilien Lasne^3,4^ & Claus Wedekind^1^*

^1^ Department of Ecology & Evolution, University of Lausanne, Lausanne, Switzerland

^2^ Swiss Institute of Bioinformatics (SIB), Environmental Bioinformatic Group, Lausanne, Switzerland

^3^ Université Savoie Mont Blanc, INRAE, MR CARRTEL, Station d’Hydrobiologie Lacustre, Thonon Cedex, France

^4^ UMR DECOD (Ecosystem Dynamics and Sustainability), INRAE, Institut Agro, IFREMER, Rennes, France

* Correspondence: Claus Wedekind, claus.wedekind@unil.ch; orcid.org/0000-0001-6143-4716

# Equal contributors

**Content**

Figure S1. Yolk-sac larvae at hatching and 14 days later

Figure S2. Larval lengths and yolk sac volumes over time

Figure S3. 1^st^ experiment: Larval size 14 dph predicted by paternal skin coloration or parental kinship

Figure S4. 2^nd^ experiment: Replotting the data and regressions presented in Figure 3 after the male with an extra-ordinary low F_β.male_ (see Figure 1) was excluded

Table S1. 1^st^ experiment: Linear mixed model on larval size measures 14 days post-hatching

Table S2. 2^nd^ experiment: Rerunning the analyses presented in Table 3 after the male with an extra-ordinary low F_β.male_ (see Figure 1) was excluded


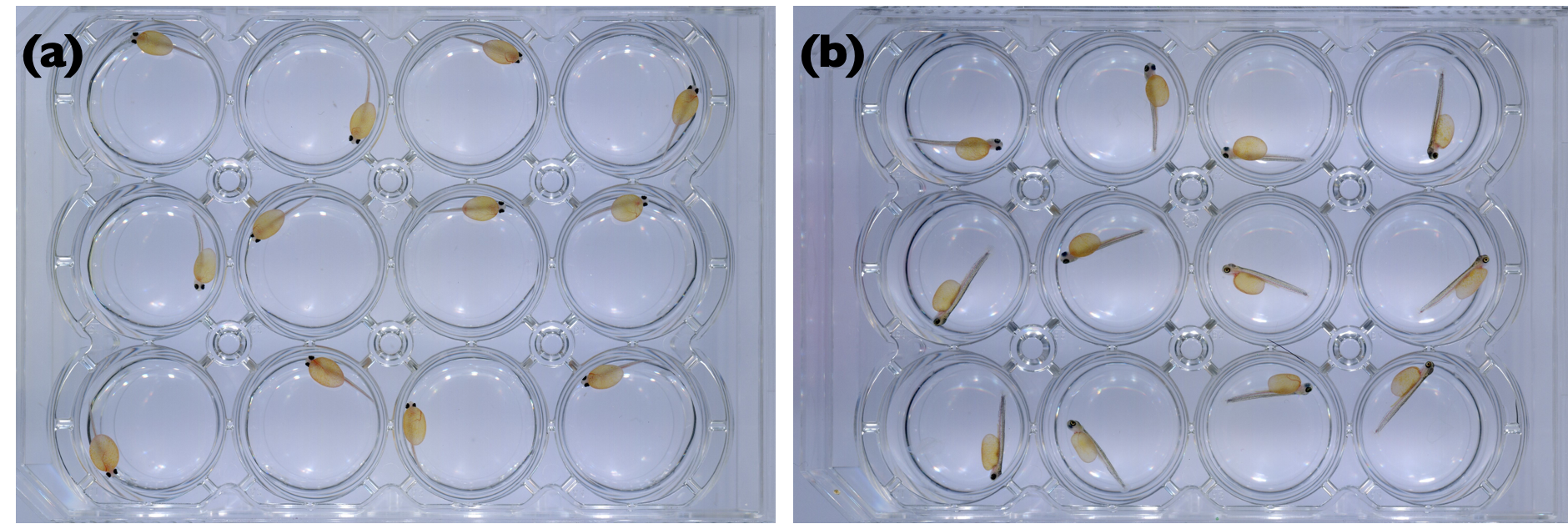


**Supplementary Figure S1.** Yolk-sac larvae raised at 4.5°C in 12-well plates from hatching on. The photos were taken (a) at hatching and (b) 14 days later.

**
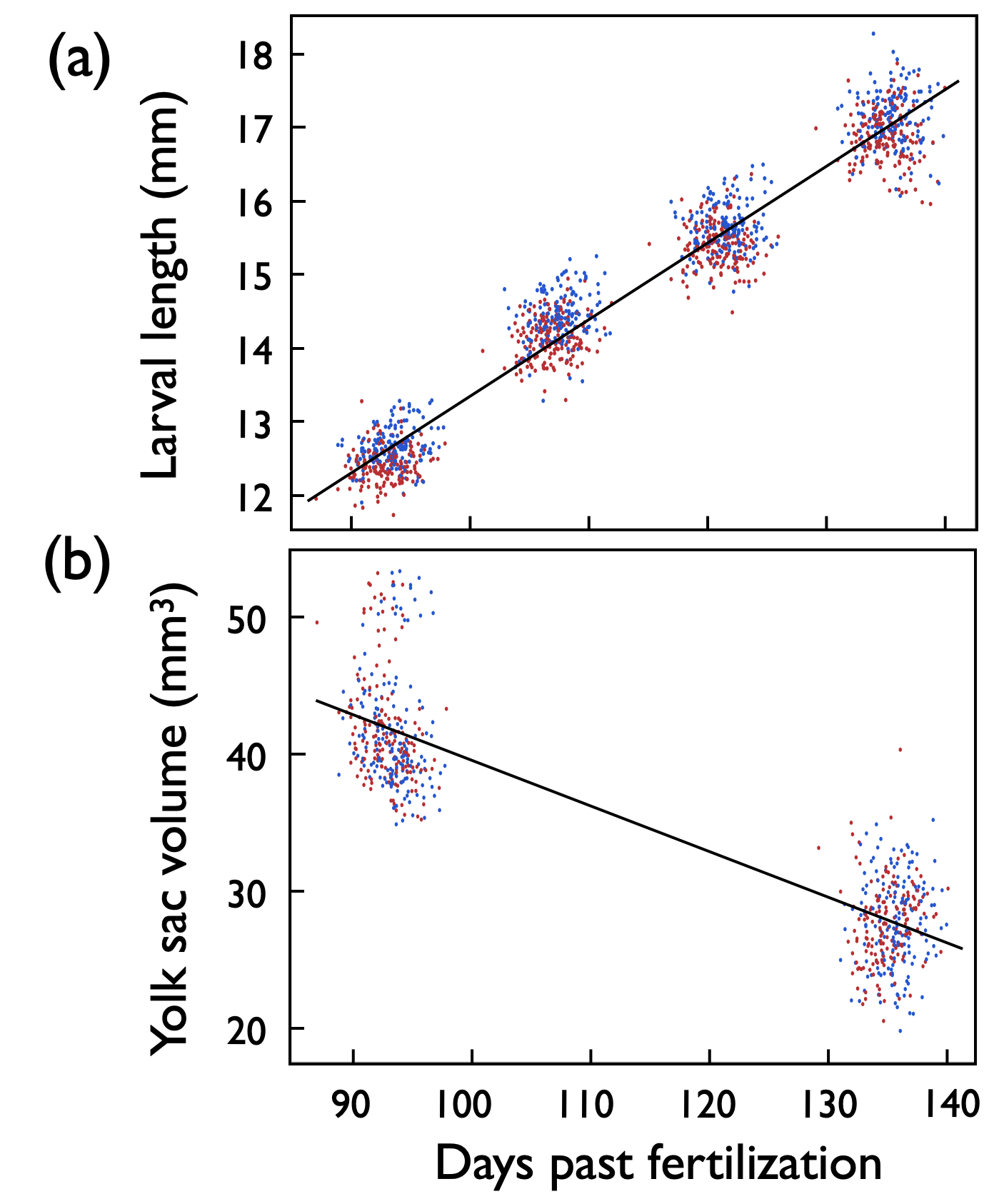
**

**Supplementary Figure S2.** Days past fertilization (dpf) versus (a) average larval length and (b) average yolk sac volume per full-sib family and treatment (red: exposed to *Aeromonas salmonicida*, blue: sham-exposed). Larval lengths were measured on the hatching day and 14, 28, and 42 days later. Yolk-sac volumes were measured on the day of hatching and 42 days later. The lines give the linear regressions.

**
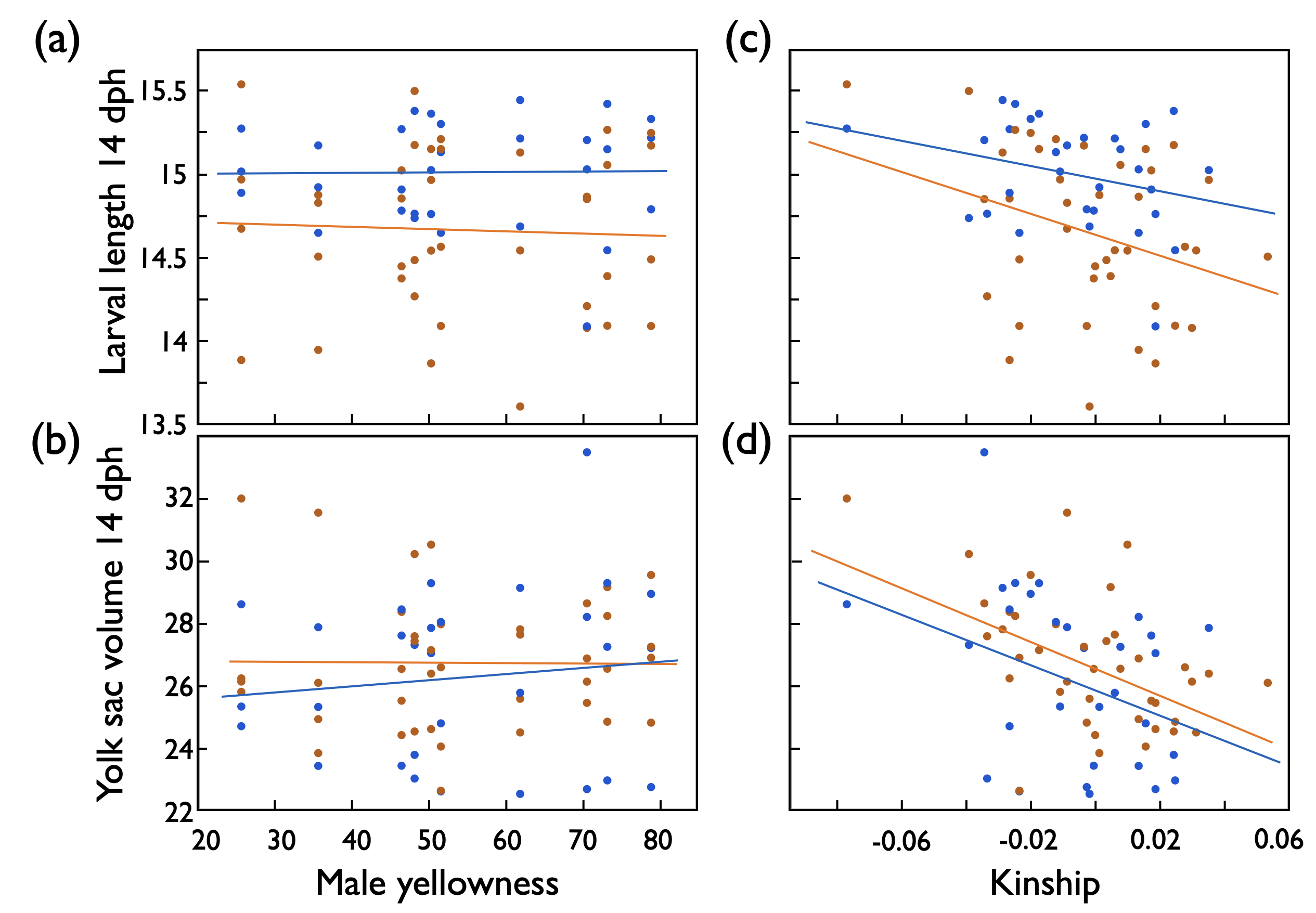
**

**Supplementary Figure S3.** Larval growth in 1^st^ experiment: Mean larval length (mm) 14 days past hatching (dph), and mean yolk sac volume at that time (means per full-sib family) predicted by (a,b) paternal skin coloration (“male yellowness”) or (c,d) by the parental kinship coefficients (“kinship”) after exposure to remainders of ovarian fluids (orange symbols and regression lines), or sham-exposed (blue). See Table S1 for statistics.

**
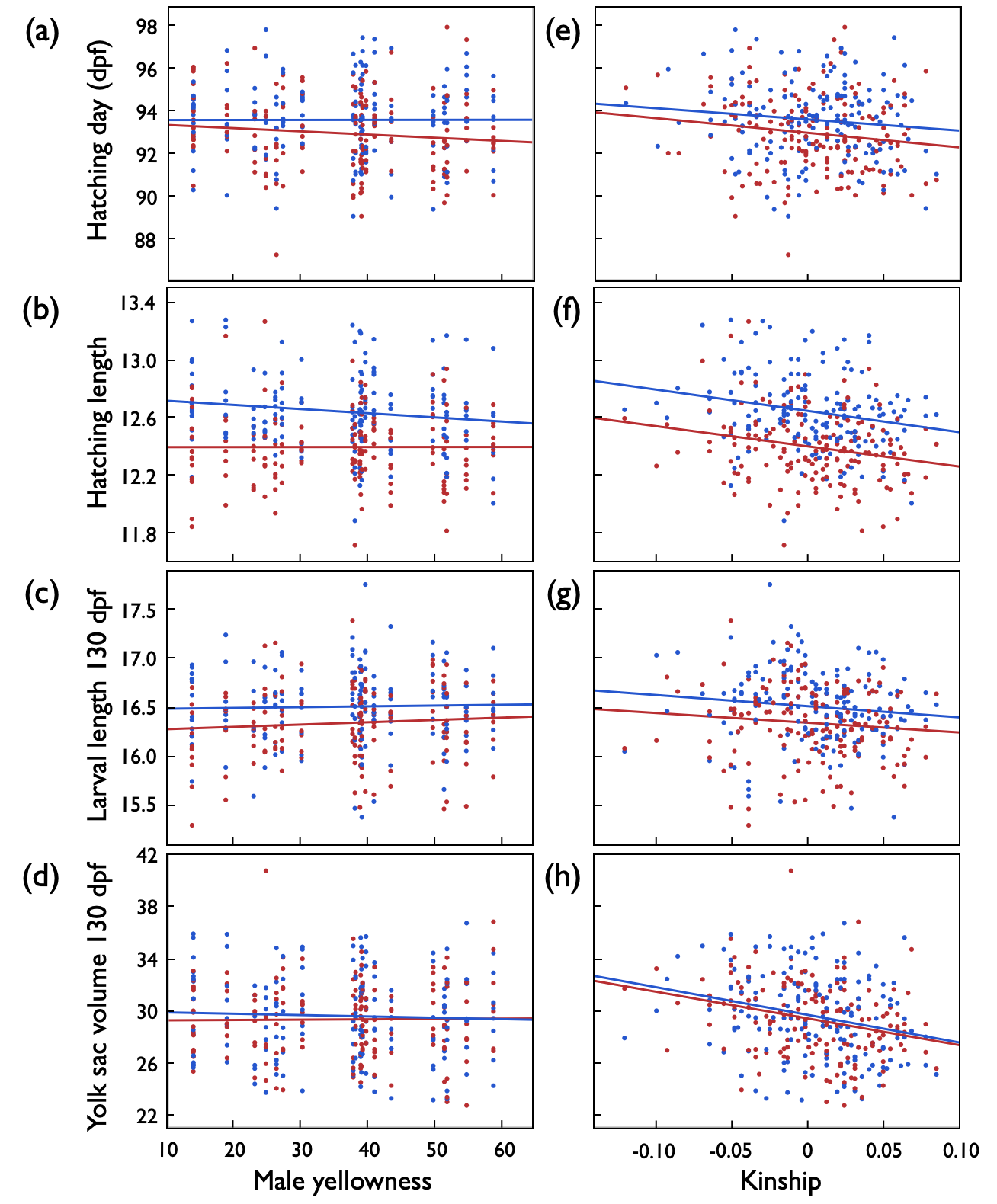
**

**Supplementary Figure S4.** Second experiment: Embryo development after exposure to *Aeromonas salmonicida* (red symbols and regression lines) or sham exposure (blue) after excluding one male with extremely low F_β.male_ and kinship values (text Figure 3). (a) Mean hatching time (dpf = days past fertilization), (b) mean larval length at hatching (mm), (c) mean larval length 130 dpf (mm), and (d) mean yolk sac volume 130 dpf (mm^3^) per full-sib family predicted by paternal skin coloration (“male yellowness”), and (e,f,g.h) predicted by the parental kinship coefficient (“kinship”). See Table S3 for statistics.

**Supplementary Table S1.** **Testing for effects on larval growth in 1^st^ experiment**: Linear mixed model on larval length and yolk sac volume 14 days post-hatching (dph) when predicted by treatment (support of microbial symbionts by remainders of ovarian fluid) and paternal skin coloration (“yellowness”) and/or the parental kinship coefficient (“kinship”), with hatching time included as a further covariate. The table gives the coefficients (± standard error), the denominator degrees of freedom (DF, based on the Kenward-Roger first-order approximation; the numerator degrees of freedom is always 1), the t values (the ratio of the coefficient to its standard error), and the corresponding two-tailed p-values. Family identity and the interaction between family ID and treatment were included as random factors. New significant p-values are highlighted in bold (parental effects and effects of treatment and hatching date had already been reported in Nusbaumer et al. (2021)).

| Effects | Larval length 14 dph | | | |  | Yolk sac volume 14 dph | | | |  | |
| --- | --- | --- | --- | --- | --- | --- | --- | --- | --- | --- | --- |
|  |  |  |  |  |  |  |  |  |  | |  |
| Fixed effects: | Coefficients | DF | t | p |  | Coefficients | DF | t | p | |  |
| Intercept | 6.48±1.38 | 1247 | 4.69 | <0.001 |  | 102.98±8.33 | 938.3 | 12.35 | <0.001 | |  |
| Treatment | 0.15±0.04 | 33.06 | 4.09 | <0.001 |  | -0.35±0.17 | 25.88 | -2.05 | 0.05 | |  |
| Yellowness | 0.0004±0.004 | 37.37 | 0.10 | 0.92 |  | 0.001±0.015 | 30.23 | 0.65 | 0.52 | |  |
| Kinship | -5.37±3.01 | 40.14 | -1.78 | 0.08 |  | -43.4±10.8 | 34.69 | -4.02 | **<0.001** | |  |
| Yellowness x kinship | -0.10±0.16 | 39.55 | -0.60 | 0.55 |  | -0.63±0.58 | 36.06 | -1.09 | 0.28 | |  |
| Treatment x yellowness | <0.001±0.002 | 30.9 | 0.02 | 0.98 |  | 0.01±0.01 | 23.24 | 1.07 | 0.30 | |  |
| Treatment x kinship | 1.56±1.55 | 37 | 1.01 | 0.32 |  | 4.17±7.17 | 31.54 | 0.58 | 0.56 | |  |
| Hatching time | 0.09±0.02 | 1321 | 6.13 | <0.001 |  | -0.86±0.09 | 986.5 | -9.35 | <0.001 | |  |
|  |  |  |  |  |  |  |  |  |  | |  |
| Random effects^1^: | Variance components |  |  |  |  | Variance components |  |  |  | |  |
| Family ID | 0.13±0.04 |  |  | 0.002 |  | 1.06±0.61 |  |  | 0.08 | |  |
| Family x treatment | 0.05±0.02 |  |  | 0.03 |  | 0.32±0.48 |  |  | 0.50 | |  |
| Residual | 0.65±0.03 |  |  |  |  | 26.79±1.06 |  |  |  | |  |

^1^ REML unbounded variance components ± standard error, Wald p-values

**Supplementary Table S2.** Second experiment: Rerunning the analyses presented in Table 3 when excluding the male with an extra-ordinary low F_β.male_ (see Figure 1): Linear mixed model on embryo hatching time, hatchling length, and larval length and yolk sac volume at 130 dpf when predicted by treatment (exposure to *Aeromonas salmonicida*) and paternal skin coloration (“yellowness”) and/or the parental kinship coefficient (“kinship”). Hatching time was included as a further covariate when testing for effects on hatchling length. The table gives the coefficients (± standard error), the denominator degrees of freedom (DF, based on the Kenward-Roger first-order approximation; the numerator degrees of freedom is always 1), the t values (the ratio of the coefficient to its standard error), and the corresponding two-tailed p-values. Significant p-values are highlighted in bold.

| Effects | Hatching time | | | | Hatchling length | | | | Larval length 130dpf | | | | | Yolk sac volume 130dpf | | | | | | |
| --- | --- | --- | --- | --- | --- | --- | --- | --- | --- | --- | --- | --- | --- | --- | --- | --- | --- | --- | --- | --- |
|  |  |  |  |  |  |  |  |  |  |  |  |  |  | |  |  |  |  | |  |
| Fixed effects: | Coefficients | DF | t | p | Coefficients | DF | t | p | Coefficients | DF | t | p | Coefficients | | DF | t | p |  | |  |
| Intercept | 93.55±0.36 | 164.2 | 257.6 | **<0.001** | 7.11±0.32 | 2527 | 22.0 | **<0.001** | 21.42±0.43 | 2154 | 49.3 | **<0.001** | -27.30±4.41 | | 2282 | -6.19 | **<0.001** | |  | |
| Treatment | -0.31±0.07 | 153.9 | -4.59 | **<0.001** | -0.11±0.01 | 158.3 | -12.9 | **<0.001** | -0.10±0.01 | 160.8 | -10.4 | **<0.001** | 0.06±0.11 | | 156 | 0.56 | 0.57 | |  | |
| Yellowness | -0.008±0.01 | 163.7 | -0.82 | 0.41 | -0.0001±0.001 | 159.8 | -0.08 | 0.94 | 0.002±0.002 | 158.4 | 1.12 | 0.26 | 0.01±0.02 | | 163.3 | 0.83 | 0.41 | |  | |
| Kinship | -4.74±3.28 | 162.6 | -1.45 | 0.15 | -0.86±0.46 | 158.2 | -1.89 | 0.06 | -1.45±0.64 | 157.1 | -2.28 | **0.02** | -19.33±5.39 | | 162.4 | -3.59 | **<0.001** | |  | |
| Yellowness x kinship | 0.40±0.28 | 165.2 | 1.43 | 0.16 | 0.03±0.04 | 161.5 | 0.69 | 0.49 | 0.04±0.05 | 160.4 | 0.67 | 0.50 | -0.33±0.46 | | 165.6 | -0.73 | 0.47 | |  | |
| Treatment x yellowness | -0.007±0.005 | 152.6 | -1.22 | 0.22 | 0.002±0.001 | 152.3 | 2.77 | **0.006** | 0.0001±0.0008 | 152.7 | 0.13 | 0.89 | 0.01±0.01 | | 147.7 | 1.33 | 0.19 | |  | |
| Treatment x kinship | -0.30±1.82 | 151.6 | -0.17 | 0.87 | -0.05±0.22 | 150 | -0.22 | 0.83 | -0.03±0.27 | 153.7 | -0.13 | 0.90 | -0.18±2.82 | | 147 | -0.06 | 0.95 | |  | |
| Hatching time |  |  |  |  | 0.06±0.003 | 2624 | 17.0 | **<0.001** | -0.05±0.005 | 2184 | -11.9 | **<0.001** | 0.60±0.05 | | 2351 | 12.91 | **<0.001** | |  | |
|  |  |  |  |  |  |  |  |  |  |  |  |  |  | |  |  |  | |  | |
| Random effects^1^: | Variance components |  |  |  | Variance components |  |  |  | Variance components |  |  |  | Variance components | |  |  |  | |  | |
| Family ID | 1.55±0.27 |  |  | **<0.001** | 0.03±0.005 |  |  | **<0.001** | 0.07±0.01 |  |  | **<0.001** | 4.27±0.71 | |  |  | **<0.001** | |  | |
| Family x treatment | 1.05±0.17 |  |  | **<0.001** | 0.004±0.002 |  |  | 0.10 | 0.003±0.003 |  |  | 0.37 | 0.51±0.40 | |  |  | 0.20 | |  | |
| Residual | 4.13±0.11 |  |  |  | 0.16±0.004 |  |  |  | 0.20±0.006 |  |  |  | 24.27±0.71 | |  |  |  | |  | |

^1^ REML unbounded variance components ± standard error, Wald p-values
